# Genomic selection using low density marker panels with application to a sire line in pigs

**DOI:** 10.1186/1297-9686-45-28

**Published:** 2013-07-29

**Authors:** Robin Wellmann, Siegfried Preuß, Ernst Tholen, Jörg Heinkel, Klaus Wimmers, Jörn Bennewitz

**Affiliations:** 1 Institute of Animal Husbandry and Animal Breeding, University of Hohenheim, D-70599 Stuttgart, Germany; 2 Institute of Animal Science, Animal Breeding and Husbandry Group, University of Bonn, D-53115 Bonn, Germany; 3 Landesanstalt für Schweinezucht, Seehöfer Straße 50, D-97944, Boxberg–Windischbuch, Germany; 4 Leibniz Institute for Farm Animal Biology (FBN), Wilhelm-Stahl Allee 2, D-18196, Dummerstorf, Germany

## Abstract

**Background:**

Genomic selection has become a standard tool in dairy cattle breeding. However, for other animal species, implementation of this technology is hindered by the high cost of genotyping. One way to reduce the routine costs is to genotype selection candidates with an SNP (single nucleotide polymorphism) panel of reduced density. This strategy is investigated in the present paper. Methods are proposed for the approximation of SNP positions, for selection of SNPs to be included in the low-density panel, for genotype imputation, and for the estimation of the accuracy of genomic breeding values. The imputation method was developed for a situation in which selection candidates are genotyped with an SNP panel of reduced density but have high-density genotyped sires. The dams of selection candidates are not genotyped. The methods were applied to a sire line pig population with 895 German Piétrain boars genotyped with the PorcineSNP60 BeadChip.

**Results:**

Genotype imputation error rates were 0.133 for a 384 marker panel, 0.079 for a 768 marker panel, and 0.022 for a 3000 marker panel. Error rates for markers with approximated positions were slightly larger. Availability of high-density genotypes for close relatives of the selection candidates reduced the imputation error rate. The estimated decrease in the accuracy of genomic breeding values due to imputation errors was 3% for the 384 marker panel and negligible for larger panels, provided that at least one parent of the selection candidates was genotyped at high-density.

Genomic breeding values predicted from deregressed breeding values with low reliabilities were more strongly correlated with the estimated BLUP breeding values than with the true breeding values. This was not the case when a shortened pedigree was used to predict BLUP breeding values, in which the parents of the individuals genotyped at high-density were considered unknown.

**Conclusions:**

Genomic selection with imputation from very low- to high-density marker panels is a promising strategy for the implementation of genomic selection at acceptable costs. A panel size of 384 markers can be recommended for selection candidates of a pig breeding program if at least one parent is genotyped at high-density, but this appears to be the lower bound.

## Background

Genomic selection refers to the use of large numbers of single nucleotide polymorphisms (SNPs) spread across the genome for breeding value estimation and subsequent selection of individuals based on genomically enhanced breeding values [1,2]. This technique has become a standard tool in dairy cattle breeding schemes, where it shortens the generation interval substantially [3]. The benefits for other livestock species like pigs or sheep are less obvious, mainly because generation intervals are already small and there is not so much scope for further reduction. However, Simianer [4] and Lillehammer et al. [5] showed that genomic selection can also be relevant in pig breeding schemes. With genomic selection, it is possible to exclude some selection candidates from progeny testing, and to increase selection intensities and the accuracy of the breeding values. Indeed some pig organisations have started to implement this technique. In Germany, for example, sire line pig breeding is dominated by Piétrain herdbook associations that apply sire progeny testing on stations for growth, carcass and meat quality traits. Some stations have started to implement genomic selection by genotyping these progeny tested sires with the Illumina PorcineSNP60 BeadChip [6] and to use them as the initial reference population [7]. From an economical point of view, the most critical point is the high cost of genotyping because individuals of the reference population and the selection candidates need to be genotyped.

One way to reduce routine costs is to genotype selection candidates with an SNP panel of reduced density [8-10]. Missing genotypes can then be imputed using genotyping information from the individuals in the reference population and the genomic breeding values can be estimated for the selection candidates in the same way as if they were genotyped for the full set of SNPs. The accuracy of imputation depends on several factors, such as the number of SNPs in the low density panel, their informativeness and distribution across the genome, the relationship between the animals genotyped, the effective population size, and the method used. Livestock species usually have a low effective population size and a limited number of founder genome equivalents [11]; both characteristics are helpful in imputing missing genotypes. Various imputation methods are available, some of which are reviewed in [12]. In general, they can be classified based on whether they use linkage disequilibrium (LD), e.g. fastPHASE [13] and Beagle [14], or pedigree information [8,15]. Some methods use both types of information, e.g. LDMIP [16] and AlphaImpute [17].

Many SNPs of the available chips have unknown chromosomal positions. For example, the PorcineSNP60 BeadChip contains > 62 000 SNP, of which 30% have no known position on the porcine genome sequence (build 7 version) [6]. In the current assembly of the pig genome (build 10.2), which was used in this study, the proportion of SNPs with an unknown position was reduced. The SNP position is not crucial for genomic breeding value estimation, but it is needed if genotype imputation is performed. One obvious solution is to exclude SNPs with unknown positions but this may cause a substantial loss of genotypic information. Alternatively, the position could be estimated from the experimental data in a ‘classical’ way using linkage analysis. Since an approximated position might be sufficient for genotype imputation, the positions could also be approximated using LD information.

The aim of the present study was to evaluate different strategies for genomic selection in a sire line pig population using low-density marker panels. The strategies included methods for the approximation of SNP positions, for the selection of SNPs to be included in the low-density panel, for genotype imputation, and for the estimation of the accuracy of genomic breeding values. The methods were validated using genotype imputation error rate, imputation accuracy, correlation between direct genomic values (DGV) and BLUP breeding values (EBV), and approximate accuracies of DGV.

## Methods

### Materials

Genotypes and EBV of 895 German Piétrain boars were available from breeding organizations. Boars were genotyped with the PorcineSNP60 BeadChip [6]. Sows were not genotyped. After removal of SNPs with a call rate less than 95%, with more than 2% parent progeny conflicts, a minor allele frequency (MAF) less than 3%, or significant departure from HWE (p < 0.0001), 48 062 markers remained and were used for the analysis. Markers with unknown physical positions were not removed but given an appropriate position, as described below. Alleles were coded as 0 and 1 such that allele 0 had the higher frequency. The three possible genotypes 0, 1, and 2 were defined as the number of copies of allele 1.

The boars were progeny tested with a varying number of offspring. Conventional EBV were available for 14 growth, carcass and meat quality traits from the routine animal evaluation centre (see Table 1). The genotyped individuals were split into a training set and a validation set, as described below. Deregressed EBV were used as records for the estimation of direct genomic values (DGV) [18].

**Table 1:** Trait names and accuracy of conventional BLUP estimated breeding values

**Trait**	**Abbreviation**	**Complete pedigrees**		**Short pedigrees**	
		**Validation**	**Training**	**Validation**	**Training**
Daily gain, field test records	DGfield	0.92	0.81	0.92	0.76
Daily gain, on station test records	DGstation	0.82	0.79	0.79	0.73
Carcass lean content, estimated with Bonner formulae	CLCBonn	0.76	0.78	0.68	0.70
Carcass lean content, FOM records	CLCFOM	0.90	0.78	0.88	0.72
Shoulder weight, AutoFOM records	SW	0.78	0.69	0.71	0.60
Belly weight, AutoFOM records	BW	0.77	0.69	0.71	0.61
Belly lean content, AutoFOM records	BLC	0.83	0.74	0.79	0.67
Ham weight, AutoFOM records	HW	0.79	0.72	0.73	0.65
Loin weight, AutoFOM records	LW	0.74	0.68	0.63	0.59
Loin eye area	LEA	0.71	0.76	0.58	0.68
Carcass length	CL	0.62	0.72	0.39	0.59
pH value, loin, 45 min. p.m.	pH1	0.60	0.70	0.25	0.52
Intramuscular fat content	IMF	0.55	0.53	0.22	0.36
Drip loss	Drip	0.59	0.67	0.24	0.50
Mean		0.74	0.72	0.61	0.62

Traits, trait abbreviations and accuracy of conventional BLUP estimated breeding values of animals in the validation and training sets, derived using complete and short pedigrees.

Two sets of EBV were calculated. For the first set, EBV were estimated using complete pedigrees. However, since the EBV of the individuals in the training and validation data sets were estimated in a single evaluation, not only the EBV but also the prediction errors of related individuals were correlated. This error correlation leads to an overestimation of accuracy if it is not accounted for [19]. This is a problem, especially if the accuracies of the EBV are low due to a limited number of offspring, which is the case in pig breeding. In order to examine the extent to which the error correlation led to an overestimation of accuracies, a second set of EBV was calculated. For the second set, the parents of the high-density genotyped boars were considered unknown, so the parental averages did not contribute to their EBV. This shortened pedigree was used to avoid correlations between prediction errors of the EBV of the genotyped boars. Traits and accuracies of the EBV are shown in Table 1.

The validation set for both imputation and genomic selection consisted of 100 boars with genotyped sires but without genotyped offspring. The remaining 795 boars were included in the training set. Thus, the sire (but not the dam) of every individual from the validation set was in the training set. The individuals of the validation set were chosen from the latest delivered genotypes. In order to avoid a possible bias due to overfitting the model, we excluded these 100 boars (i.e. their EBV and their genotypes) from all evaluations done during the development of the imputation method.

### LD-based position approximation of markers with unknown positions

In order to also use markers with unknown physical position for imputation, their positions were approximated using LD information, as described below. Let **G**_*m*_ to be the vector of genotypes of the individuals for marker *m* and *MAF*_*m*_ the MAF of this marker. We constructed equivalence classes of marker sets such that markers from the same equivalence class are expected to belong to the same chromosome. Two markers *m*′ and *m*″ belong to the same equivalence class if a chain *m*_1_,..., *m*_*n*_ of markers exists, starting with marker *m*_1_ = *m*′ and ending with marker *m*_*n*_ = *m*″, such that the absolute value of the correlation of the genotypes for adjacent markers in the chain is larger than a threshold value of 0.4. That is,

$$\begin{array}{ccc} \left|\mathrm{Cor}\left(G_{m_{k}},G_{m_{k+1}}\right)\right|>0.40 & \mathrm{for} & k=1,...,n-1, \end{array}$$

with $\mathrm{MA}F_{m_{k}}>0.15$ for *k* = 2,..., *n* − 1. The threshold value 0.4 was chosen to avoid equivalence classes to cover multiple chromosomes. Correlations of genotypes (coded 0,1,2) were used and not the LD measure *r*[20], because haplotypes are unknown for markers with unknown positions. If for a given equivalence class more than 95% of the markers with known physical positions belonged to the same chromosome, then all markers in this equivalence class were mapped to that chromosome, resulting in the assignment of markers with unknown positions to chromosomes. In order to enable imputation, the position of a marker *m*^′^ with unknown position on the identified chromosome was then set equal to the known position of a marker *m* from the same chromosome that maximized $\left|\mathrm{Cor}\left(G_{m},G_{m^{\prime }}\right)\right|$. Using this LD-based method, the position could be approximated for 3930 markers; positions of 153 markers remained unknown. Moreover, 336 markers with position information based on build 10.2 were mapped to a different chromosome than originally assigned and these new positions were used in the present study.

### Construction of low-density panels

From the set of markers, four subsets consisting of 384, 2 · 384 = 768, 3 · 384 = 1152 and 3000 SNPs were selected based on equidistant location, high MAF, and low correlation of genotypes, as described in the following three steps. Let *loc*_*m*_ be the location in megabases (Mb) and *Chr*_*m*_ the chromosome of SNP *m*. In the first step, we defined a distance measure *d* between two SNPs *m′* and *m*″ as *d*(*m*′, *m*″) = ∞ if *Chr*_*m*′_ ≠ *Chr*_*m*″_, and if *Chr*_*m*′_ = *Chr*_*m*″_ then

$$\begin{array}{l} d\left(m\prime ,m\prime \prime \right)=\lambda \left|\mathrm{lo}c_{m\prime }-\mathrm{lo}c_{m\prime \prime }\right|\\ \;+\left(1-\lambda \right)\kappa \left(1-0.99\cdot \left|\mathrm{Cor}\left(G_{m\prime },G_{m\prime \prime }\right)\right|\right), \end{array}$$

where $\lambda =\min\left(1,\frac{\left|\mathrm{lo}c_{m\prime }-\mathrm{lo}c_{m\prime \prime }\right|}{\kappa }\right)$, with *κ* = 5. Following this, we have *λ* < 1 (*λ* = 1) if the markers were separated by less than (more than) *κ* = 5 Mb. Hence, for closely linked loci (*λ* < 1), the correlation between the genotypes contributed to the distance measure. This was done so that two markers at similar genomic positions could be included in the low-density panel if they were not in LD and, hence, the correlation between their genotypes was low.

In the next step, a score was calculated for each marker *m* based on its MAF (*MAF*_*m*_):

$$\mathrm{Scor}e_{m}=\mathrm{MA}F_{m}u_{m},$$

where *u*_*m*_ = 1 if marker *m* had a known physical position in build 10.2 and if the position did not change during the editing of the marker map, and *u*_*m*_ = 0.8 if the position of the marker was estimated as described in the previous section, i.e. the latter markers were penalised in the construction of the low-density panels. In the third step, markers were selected based on their scores and the distance measure *d*. If *n* markers were already included in the low-density panel, then marker *m*_*n*+1_ was chosen such that $\mathrm{Scor}e_{m_{n+1}}\cdot \min\left(d\left(m_{n+1},m_{k}\right):k=1,...,n\right)$ was maximized, i.e. it simultaneously had a high score and was at a large distance to all previously chosen markers. Roughly speaking, marker *m*_*n*+1_ was chosen from the largest gap, provided that a marker in the gap had a high score.

For comparison, we also considered a naïve approach in which all markers had the same score (*Score*_*m*_ = 1), *κ* was close to zero, and only markers with known physical positions were included. This resulted in low-density panels with approximately equally spaced markers since they were subsequently chosen from the centre of the largest gap.

### Phasing of high-density genotypes and imputation of genotypes from low- to high-density

The high-density genotypes of all individuals in the training set were phased with Beagle version 3.3.2 by declaring individuals as unrelated or by including them as parent-offspring pairs. The algorithm is iterative and described in detail in [14,21,22]. Following the phasing step, imputation from low- to high-density genotypes was done using a novel method that assumed that the sires of all low-density genotyped individuals were genotyped at high-density, as is probably the case in breeding schemes applying genomic selection with low-density panels. Imputation was done as follows. For each low-density genotyped individual, the imputation algorithm tried to determine whether the individual inherited the paternal or the maternal allele of the sire for all low-density markers. This is possible with certainty if the individual is homozygous at marker *m* and the sire is heterozygous. Let *Which*_*m*_ = 0 if the individual inherited the paternal allele of the sire at marker *m*, *Which*_*m*_ = 1 if it inherited the maternal allele, and *Which*_*m*_ = −1 if the origin could not be determined. Thereafter, the origins of the remaining paternal alleles of the individual were estimated. Let *m*_1_ and *m*_2_ be low-density markers from the same chromosome for which the origin of the paternal allele could be determined and assume that the origin could not be determined for all low-density markers between *m*_1_ and *m*_2_. If the paternal alleles at markers *m*_1_ and *m*_2_ have the same origin ($\mathrm{Whic}h_{m_{1}}=\mathrm{Whic}h_{m_{2}}$), then it is assumed that all paternal alleles between *m*_1_ and *m*_2_ also have this origin ($\mathrm{Whic}h_{m}=\mathrm{Whic}h_{m_{1}}$ for all *m*_1_ < *m* < *m*_2_). If the alleles have a different origin ($\mathrm{Whic}h_{m_{1}}\neq \mathrm{Whic}h_{m_{2}}$), then it is assumed the cross-over occurred at the centre of the interval and paternal alleles between $\mathrm{lo}c_{m_{1}}$ and $\frac{\mathrm{lo}c_{m_{1}}+\mathrm{lo}c_{m_{2}}}{2}$ were assigned the same origin as *m*_1_, and paternal alleles between $\frac{\mathrm{lo}c_{m_{1}}+\mathrm{lo}c_{m_{2}}}{2}$ and $\mathrm{lo}c_{m_{2}}$ were assigned the same origin as *m*_2_.

**Figure 1:**
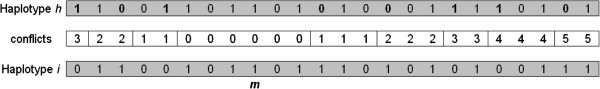
**Illustration of the definition of**$c_{h,i}^{m}\left(k\right)$**for imputation of maternally inherited alleles.** Haplotype *i* is the maternal haplotype of the individual; haplotype *h* is one of the haplotypes from the haplotype library that is to be scored; for a specified value of *k* (*k* = 0, 1, 2, 3, 4), the number $c_{h,i}^{m}\left(k\right)$ of markers for which there were exactly *k* haplotype conflicts in the interval between the respective marker allele and marker *m* was calculated; with respect to marker *m*, the number of markers with *k* = 0 conflict is $c_{h,i}^{m}\left(0\right)=6$; for *k* = 1, 2, 3, 4, the numbers of markers with *k* conflicts are $c_{h,i}^{m}\left(1\right)=5$, $c_{h,i}^{m}\left(2\right)=5$, $c_{h,i}^{m}\left(3\right)=3$, and $c_{h,i}^{m}\left(4\right)=3$, respectively.

Thereafter, the paternal alleles of the individual for the high-density SNPs were imputed from their origin and the maternal alleles for the low-density markers were determined. The remaining maternal alleles were imputed using the haplotype library obtained from Beagle because it was assumed that the dams were not genotyped. Maternal alleles were determined as described below. For each low-density marker *m*, the haplotypes of all high-density genotyped individuals were scored and the haplotype with the highest score was imputed over the largest range for which there was no allele conflict with the maternal haplotype *i* of the individual. Let *h* be the haplotype that is to be scored. The score of haplotype *h* at marker *m* is defined as

$$\mathrm{Scor}e_{i,m}\left(h\right)={\left(\sum_{k=0}^{4}c_{h,i}^{m}\left(k\right)\cdot 0.75^{k}\right)}^{1+a_{i,h}},$$

where $c_{h,i}^{m}\left(k\right)$ is the number of low-density markers $\tilde{m}$ for which exactly *k* low-density markers between *m* and $\tilde{m}$ have different alleles at haplotypes *h* and *i*. The definition of $c_{h,i}^{m}\left(k\right)$ is illustrated in Figure 1. Inclusion of summands for *k > 0* was done to make the score more robust with respect to phasing errors. The parameter *a*_*i*,*h*_ is the additive genetic relationship between the mother of the individual to be imputed and the individual with haplotype *h*, which was calculated from the pedigree. In particular, *a*_*i*,*h*_ = 0.5 if the individual with haplotype *h* is the maternal grand sire and *a*_*i*,*h*_ = 0.25 if the individual with haplotype *h* is a maternal great grand sire. The results obtained with this imputation method were compared with the results obtained from Beagle.

### Estimation of genomic breeding values

Direct genomic breeding values were estimated with GBLUP [1] from the deregressed EBV. GBLUP was extended to account for heterogeneous error variances. Based on [18], the error variance for individual *i* was proportional to $V_{A}\left(C+\frac{1-r_{\mathrm{EBV}}^{2}\left(i\right)}{r_{\mathrm{EBV}}^{2}\left(i\right)}\right)$, where *C* is the fraction of the additive variance not explained by markers and $r_{\mathrm{EBV}}^{2}\left(i\right)$ is the reliability of the EBV of individual *i*. We assumed *C* = 0.25.

### Estimation of imputation accuracy

The imputation error rate and imputation accuracies were calculated for each marker panel and method. SNPs not included in the low-density panels were masked in the validation set and imputed using the training set. The imputation error rate was computed as the proportion of masked SNP genotypes that were not correctly imputed. The imputation accuracy for an individual was computed as the squared correlation between its true and imputed genotypes. To avoid that the coding of the markers affected the imputation accuracy, every marker was considered twice. The first time the alleles were coded as 0 and 1, and the second time, the numbers were interchanged.

### Estimation of DGV accuracies

The individuals were divided into a training set and a validation set as described above. For individuals in the validation set, the correlation *cor*(*DGV*, *EBV*)_*t*_ between DGV and EBV was estimated for each trait *t*. The parameter of most interest, however, is the correlation between DGV and true breeding values (TBV), which will be referred to as the accuracy of the DGV. Since the breeding values for individuals in the training set and the validation set were taken from the same genetic evaluation, there is a substantial correlation of prediction errors among individuals for the EBV that were computed using the complete pedigree. Thus, the frequently used formula to derive the accuracy of DGV, i.e.

(1) $$\mathrm{cor}\left(\mathrm{DGV},\mathrm{TBV}\right)=\frac{\mathrm{cor}\left(\mathrm{DGV},\mathrm{EBV}\right)}{\mathrm{cor}\left(\mathrm{TBV},\mathrm{EBV}\right)}$$

is expected to provide estimates that are substantially biased upwards [19]. Hence, another approach was needed and we used the following regression methodology.

Suppose that for *n* randomly chosen traits, the correlation *cor*(*DGV*, *EBV*)_*t*_ has been estimated. These estimates have been obtained for different mean accuracies $r_{t}^{\mathrm{Val}}$ and $r_{t}^{\mathrm{Train}}$ of the EBV in the validation set and the training set, respectively. We predicted the expected correlation between EBV and DGV of a randomly chosen trait by assuming the following linear regression model

(2) $$\mathrm{cor}{\left(\mathrm{DGV},\mathrm{EBV}\right)}_{t}=a_{0}+a_{1}r_{t}^{\mathrm{Val}}+a_{2}r_{t}^{\mathrm{Train}}+e_{t}$$

where the intercept *a*_0_ and the regression coefficients *a*_1_ and *a*_2_ are fixed effects, and the errors *e*_*t*_ are normally distributed. Note that for $r_{t}^{\mathrm{Val}}\to 1$, the EBV approximates the TBV, so the contribution of the prediction error to the correlation approaches zero. Thus, for $r_{t}^{\mathrm{Val}}=1$, the expected correlation between EBV and DGV equals the expected accuracy of DGV for a randomly chosen trait with $r_{t}^{\mathrm{Train}}$ specified. This was estimated as

$$\hat{c}\mathrm{or}{\left(\mathrm{DGV},\mathrm{TBV}\right)}_{\mathrm{rand}}={\hat{a}}_{0}+{\hat{a}}_{1}\cdot 1+{\hat{a}}_{2}\cdot r_{t}^{\mathrm{Train}}.$$

For simplicity we assumed that possible dependency of the error *e*_*t*_ on $r_{t}^{\mathrm{Val}}$ is negligible. In this case, the accuracy of DGV for trait *t* can be estimated as

(3) $$\begin{array}{c} \hat{c}\mathrm{or}{\left(\mathrm{DGV},\mathrm{TBV}\right)}_{t}={\hat{a}}_{0}+{\hat{a}}_{1}\cdot 1+{\hat{a}}_{2}\cdot r_{t}^{\mathrm{Train}}+{\hat{e}}_{t}\\ \;=\mathrm{cor}{\left(\mathrm{DGV},\mathrm{EBV}\right)}_{t}-{\hat{a}}_{1}{\left(r_{t}^{\mathrm{Val}}-1\right)}^{.} \end{array}$$

## Results

Table 2 shows the imputation error rate and the imputation accuracies for the different marker panels and imputation methods. Error rates for SNPs with estimated physical locations were slightly larger than error rates for SNPs with known locations, but the error rates of both types of SNPs decreased as the size of the low-density panel increased. The error rate for SNPs with estimated positions was only 0.029 for the 3000 marker panel. Imputation with Beagle produced larger error rates than our imputation method, especially for very low-density panels. This shows that using information on relatives is important to achieve an acceptable error rate with very low-density marker panels. As expected, a low-density panel with equally spaced markers resulted in larger error rates than a panel in which markers with high MAF were favoured.

**Table 2:** Genotype imputation error rate and imputation accuracy for different methods

**Phasing**	**Imputation method**	**Marker selection**	**Marker position**	**Number of markers in low-density panel**			
				**384**	**768**	**1152**	**3000**
Beagle	This paper	large MAF	known	0.133/0.79	0.079/0.87	0.054/0.91	0.022/0.96
			estimated	0.148/0.76	0.095/0.84	0.066/0.89	0.029/0.95
Beagle	Beagle	large MAF	known	0.263/0.56	0.140/0.76	0.088/0.85	0.027/0.95
			estimated	0.283/0.52	0.165/0.71	0.107/0.82	0.036/0.94
Beagle	This paper	equally spaced	known	0.164/0.74	0.110/0.82	0.085/0.86	0.037/0.94
			estimated	0.183/0.70	0.128/0.79	0.101/0.83	0.050/0.92

Genotype imputation error rate and imputation accuracy (error/accuracy) for different sizes of low-density marker panels using two imputation methods for markers with known and estimated chromosomal positions.

Table 3 shows the influence of marker information from relatives on imputation error rate and imputation accuracy. For 73 individuals from the validation set, the sire (S) and the maternal grand sire (GS) were genotyped (column S + GS). For 27 individuals in the validation set, the GS was not genotyped (column S). The error rate was smaller if the grand sire was genotyped at high-density. The error rate for markers decreased from 0.149 to 0.129 for the 384 marker panel if GS was genotyped, and decreased from 0.027 to 0.022 for the 3 k panel. The decrease was relatively small because other high-density genotyped relatives also contribute to the accuracy.

**Table 3:** Effect of genotyping the maternal grandsires at high-density

**Number of markers in low-density panel**	**Genotype error rate/imputation accuracy**		**Standard deviation of error rate**	
	**S**	**S + GS**	**S**	**S + GS**
384	0.149/0.77	0.129/0.79	0.036	0.026
768	0.093/0.85	0.076/0.88	0.027	0.022
1152	0.062/0.90	0.053/0.91	0.021	0.016
3000	0.027/0.96	0.022/0.96	0.013	0.007

Genotype error rate, imputation accuracy, and the standard deviation of imputation error rate for individuals with only the sire (S), or sire and maternal grandsire (S + GS) genotyped at high-density, for different low-density marker panels

**Figure 2:**
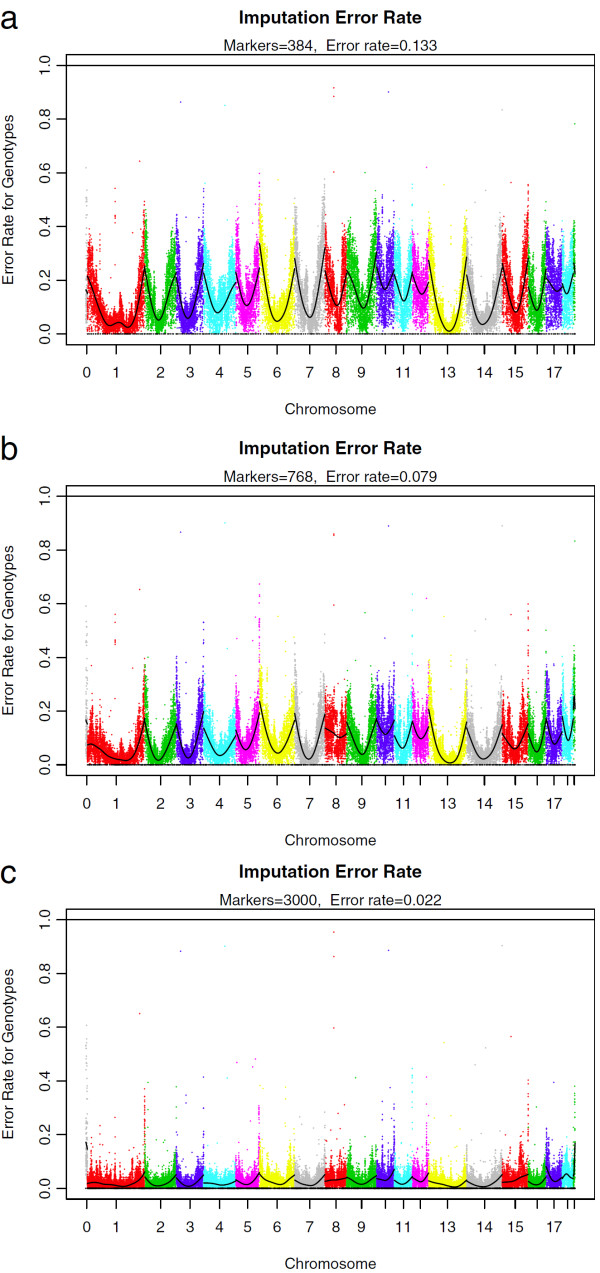
**Imputation error rate for low-density panels with a) 384 markers, b) 768 markers, and c) 3000 markers plotted against chromosomal position.** For each chromosome, a spline is plotted to illustrate the trend in the imputation error rate along the chromosome; error rates for markers on different chromosomes are shown in different colours; markers on the X chromosome are on the right hand side; the positions of the markers from the low-density panel are indicated by black points on the x-axis. The labels show for every panel the number of markers and the mean error rate of markers with known position.

Figure 2 shows the imputation error rates for masked markers. Markers in the centre of the chromosomes could be imputed with the highest accuracy. Possible reasons are that the recombination rate is higher at the chromosome ends [23], or that imputation works best if multiple low-density markers are present on both sides of an imputed marker. Since the error rate was higher at the chromosome ends, long chromosomes were imputed with higher accuracy than short chromosomes [see Additional file 1: Table S1].

Table 4 shows the correlations between DGV and EBV for different panel sizes and the effect of assuming parents of high-density genotyped individuals to be unknown in the calculation of EBV. The correlation between DGV and EBV was averaged over traits. The loss in accuracy due to imputation was smaller than expected from the imputation error rates when complete pedigrees were used. Both our imputation method and Beagle provided high correlations for the 3 k panel but our method was superior to Beagle for very low-density panels. The negligible increase in correlations when the panel size exceeds 768 markers shows that 768 markers are sufficient for imputation but 384 markers are suboptimal.

**Table 4:** Correlations between DGV and EBV computed using complete or short pedigrees, for two imputation methods

**Number of markers in low-density panel**	**Complete pedigrees**		**Short pedigrees**	
	**Own imputation**	**Beagle**	**Own imputation**	**Beagle**
384	0.60	0.45	0.27	0.24
768	0.62	0.60	0.31	0.29
1152	0.63	0.61	0.31	0.31
3000	0.62	0.62	0.31	0.31

Average correlation across traits between genomic (DGV) and conventional BLUP estimated breeding values (EBV), computed using complete or short pedigrees, for different sizes of low-density marker panels and two imputation methods.

Table 5 shows the correlations between DGV and EBV, and the estimated accuracies of the DGV. The correlation between DGV and EBV was considerably larger when complete pedigrees were used. This could have two reasons. First, when complete pedigrees were used to compute EBV, the DGV were estimated from more accurate EBV. Second, the prediction errors of the EBV from individuals in the validation set and the training set were correlated. Thus, the high correlation between DGV and EBV with use of complete pedigrees may arise from more accurately estimated prediction errors. The correlation of the prediction errors makes standard approaches for the estimation of accuracy of DGV (Equation 1) unsuitable. Therefore, a regression approach was used to derive unbiased accuracies. The parameter estimates of equation (2) were ${\hat{a}}_{0}=0.975$, ${\hat{a}}_{1}=-0.941$, ${\hat{a}}_{2}=0.490$ when complete pedigrees were used. The effect of the accuracy of the EBV in the validation set $\left({\hat{a}}_{1}\right)$ was highly significant and negative. Thus, the smaller the accuracies of the EBV in the validation set are, the greater the overestimation of the accuracy of the DGV based on the correlation between DGV and EBV. Although the correlation between DGV and EBV was considerably smaller when the shortened pedigree was used, the estimated accuracies obtained with the regression approach were, on average, almost equal with complete and short pedigrees. Equation (1) provided slightly larger estimates than the regression approach, even if shortened pedigrees were used to calculate EBV. This could be because phenotypes of the validation progeny not only affect the EBV of their sires, but also the EBV of their maternal grandsires, which suggests that the correlation of prediction errors was not negligible for traits with EBV with very low accuracies (e.g., pH1, IMF, Drip).

**Table 5:** Correlations between EBV and DGV, and accuracies of DGV estimated with different methods

**Trait**	**Cor(DGV, EBV)**	**Cor(DGV**_**1**_**,EBV**_**1**_**)**	**Cor(DGV, TBV)**	**Cor(DGV**_**1**_**, TBV)**	**Cor(DGV**_**1**_**, TBV)**
			**Regression**	**Regression**	**Equation (1)**
DGfield	0.52	0.26	0.45	0.28	0.28
DGstation	0.57	0.27	0.40	0.32	0.34
CLCBonn	0.68	0.42	0.46	0.49	0.62
CLCFOM	0.50	0.38	0.40	0.40	0.43
SW	0.61	0.33	0.40	0.39	0.46
BW	0.52	0.24	0.30	0.30	0.34
BLC	0.60	0.37	0.44	0.41	0.47
HW	0.58	0.36	0.38	0.42	0.49
LW	0.61	0.33	0.36	0.41	0.53
LEA	0.65	0.31	0.37	0.40	0.53
CL	0.60	0.18	0.25	0.31	0.46
pH1	0.83	0.31	0.45	0.47	(1.27)
IMF	0.70	0.18	0.28	0.35	(0.82)
Drip	0.83	0.36	0.44	0.52	(1.48)
Mean across traits 1-11	0.59	0.31	0.38	0.38	0.45

The first two columns show the correlations between conventional BLUP estimated breeding values (EBV) and direct genomic breeding values (DGV); the index 1 indicates that the parents of the individuals genotyped at high-density were considered unknown in the calculation of EBV; column 3 shows the estimated accuracies of the DGV when complete pedigrees were used; the last two columns show the estimated accuracies of the DGV when short pedigrees were used; see Table 1 for full names of traits.

**Figure 3:**
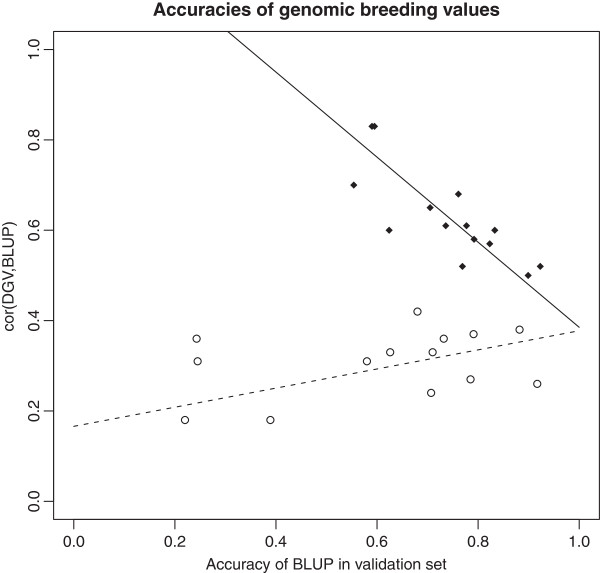
**Correlation between direct genomic values (DGV) and BLUP estimated breeding values (EBV).** The regression lines show how the correlation between DGV and EBV depends on the accuracies of the EBV in the validation set; the solid line corresponds to the situation in which complete pedigrees were used for the calculation of EBV; for the dotted line, shortened pedigrees were used.

Figure 3 visualizes the results of the regression approach. Each point in the scatter plot corresponds to one trait. The regression lines show how the correlation between DGV and EBV depends on the accuracies of the EBV in the validation set. The solid line shows the regression function $f\left(x\right)={\hat{a}}_{0}+{\hat{a}}_{2}{\bar{r}}_{\circ }^{\mathrm{Train}}+{\hat{a}}_{1}x$ when the EBV were calculated using complete pedigrees, where ${\bar{r}}_{\circ }^{\mathrm{Train}}$ is the average accuracy of the EBV in the training set. The dotted line shows the regression function that was obtained when the parents of the sires genotyped at high-density were considered unknown in the calculation of EBV (i.e. the shortened pedigrees were used). To calculate the dotted line, the three outlier traits with EBV with the lowest accuracies in the validation set were omitted.

## Discussion

### Imputation method

The results presented in this paper showed that imputation caused only a small decrease in accuracy of the DGV, even for very low-density marker panels (384 SNPs across the genome). This might be due to the performance of the genotype imputation method proposed in this study. The method uses both LD and family information. It is tailored to a situation in which at least one parent of the selection candidates is genotyped at high-density. This might be a typical situation in livestock breeding schemes that apply genomic selection with low-density panels. Compared to imputation error rates reported in most other studies, the error rates obtained in this study (Tables 2 and 3) were low. Weigel et al. [10] reported an imputation error rate of 0.29 with IMPUTE2 [24] for a low-density panel with 434 markers in Jersey cattle. In Hayes et al. [9], imputation error rates were between 0.3 and 0.4 in various sheep breeds, using a 992 marker panel and applying fastPhase [13] and were slightly higher when applying Beagle for imputation. Vereijken et al. [25] obtained a mean accuracy of imputation of about 0.75 in chickens with a low-density panel containing 384 markers and applying Beagle. However, a comparison of error rates between studies is difficult because the relationships between individuals from the training set and the validation set differ between studies, the qualities of the marker maps may differ, and the effective population sizes are not equal.

Huang et al. [26] estimated imputation accuracies in a pig population. They obtained an imputation accuracy of 0.87 on chromosome 1 with a 384 marker panel, and an imputation accuracy of 0.97 with a 3 k panel if the sire and the grand sires were genotyped at high-density (scenario s5_25% in their paper). For comparison, we obtained imputation accuracies of 0.79 and 0.96, respectively, for the whole genome (Table 3). Thus, Huang et al. [26] obtained the same results for a 3 k panel, but slightly higher accuracies for a 384 marker panel. One reason could be that we considered the complete genome. Moreover, we used Beagle for phasing, whereas Huang et al. used AlphaImpute [17], which can take pedigree information in consideration. Another reason is that we included markers with estimated positions. This explains a larger proportion of the additive variances with the high-density panel if markers with known positions are not equally spaced. However, when very low-density marker panels are used (384 markers), then it may be better to exclude markers with uncertain positions from the marker panels in order to improve imputation accuracy and phasing for the remaining markers.

For the imputation approach, all haplotypes of the haplotype library were scored for every low-density marker *m* and the haplotype *h* with the largest score was used to impute the maternal haplotype *i* of the individual in the neighbourhood of *m*. The score of a haplotype *h* depends not only on the length of the interval in which *h* coincides perfectly with haplotype *i*, but also on the mismatches at nearby SNPs. This increases the robustness of the method with respect to sporadic haplotyping errors that occur from data phasing. Moreover, inclusion of the additive relationship into the score has the effect of favouring haplotypes of closely related individuals for imputation. This is an advantage because for unrelated individuals the low-density haplotypes can by chance coincide in the neighbourhood of *m.*

Other methods such as Beagle [14], LDMIP [16], IMPUTE2 [24], ChromoPhase [15], or fastPHASE [13] can be used for imputation. These programs were, however, not well suited for the structure of the data used in our study. Beagle does not use pedigree information to impute the maternal haplotype and fastPHASE and IMPUTE2 also generally exclude pedigree information for imputation. Methods LDMIP and ChromoPhase do use pedigree information. LDMIP involves alternate multilocus iterative peeling and imputation steps. However, in the typical situation in which dams have not been genotyped, the imputation step of LDMIP fails for low-density marker panels, since the imbedded requirement of 20 markers per chromosome with a known phase is not met. The ChromoPhase algorithm alternates between a rule-based allele assignment step and a step for the identification of shared segments. This is a promising strategy that outperformed fastPHASE for simulated data [15] but as mentioned in [15], the disadvantage of this algorithm could be its sensitivity to mapping and genotyping errors.

Imputation error rates could be reduced by genotyping at high-density more close relatives of the low-density genotyped individuals (Table 3). A further reduction could be obtained by determining the correct position of markers for which the locations had only been estimated because the error rates were higher for markers with estimated positions.

### Marker selection

Markers for the low-density panels were selected based on their scores and their distances to the markers already included in the low-density panel. The MAF of the marker was used as the score. An alternative procedure for marker selection that takes MAF and distances of adjacent markers into account was proposed by Wang et al. [27]. Our approach could easily be generalized to more complex situations. Take *L*_*m*_ to be the length of the chromosome that contains marker *m*. The score may be multiplied with a factor of the form ((1 − *λ*)*L*_*m*_ + *λ* max (**L**))/*L*_*m*_ in order to ensure that more markers from the low-density panel are placed on short chromosomes. In some cases, it may be desirable that the markers from the low-density panel explain part of the additive variance. The approach can be generalized to this situation. For example, the score of a marker may be defined as its average contribution to the additive variance of traits that are standardized to have the same additive variance. That is, the score could be $\mathrm{Scor}e_{m}=2\mathrm{MA}F_{m}\left(1-\mathrm{MA}F_{m}\right)\overset{¯}{a_{m}^{2}},$ where $\overset{¯}{a_{m}^{2}}$ is the average squared estimated effect of marker *m*, averaged over all traits. Another possibility is to let the score of a marker depend on the p-values obtained from an association study, such that markers with small p-values have a higher score. It is also possible to choose more markers from the chromosome ends by modifying the distance measure.

### Accuracies of the direct genomic values

The correlations between DGV and EBV (Table 5) were substantially larger than expected for this small reference population and for the moderate reliabilities of the EBV [28-30]. One reason is that the effective number of chromosome segments is smaller for highly related individuals [31], but this observation may not fully explain the high correlations that were observed. The regression approach showed that the correlation between DGV and EBV indeed provided a strongly upwards biased estimate of the accuracy of the DGV but that this bias could be corrected for. The bias occurred because EBV of individuals in the testing and the training sets had substantial prediction error correlations. Assuming the parents of the high-density genotyped individuals to be unknown in order to reduce this error correlation had little effect on the accuracies of the DGV but reduced the correlation between DGV and EBV substantially. The latter may be considered undesirable because a high correlation between DGV and EBV increases the acceptance of the genomic predictions by the breeders. However, the relevant parameter is the accuracy of the DGV. A practical consequence of the observed effect of the accuracy of the EBV in the validation set is that increasing the size of the training population does not necessarily increase the correlation between DGV and EBV if at the same time the reliabilities of the EBV in the validation set increase. Nevertheless, increasing the size of the training population is very important to obtain accurate DGV.

The regression approach used a linear model to predict the regression function outside the range of the data. Since the linearity assumption might be violated, this approach should be evaluated in more detail with simulated data, where the true accuracies are known. The regression approach used results from multiple traits with heterogeneous accuracies of the EBV (Table 1). This approach could be transferred to a situation in which only one trait is observed, provided that EBV of the individuals have heterogeneous accuracies. In this case, a replicate *s* could be obtained by splitting the data into a training set, a validation set and an omitted set. Care should be taken so that the mean accuracies of the EBV in the validation sets vary between replicates and that the mean accuracies in the training sets are approximately equal to the average accuracy of all EBV. For these replicates the linear model $\mathrm{cor}{\left(\mathrm{DGV},\mathrm{EBV}\right)}_{s}=a_{0}+a_{1}r_{s}^{\mathrm{Val}}+e_{s}$ could be fitted, i.e. parameter *a*_2_ could be omitted if only one trait is considered.

## Conclusions

Methods for genomic selection using low-density marker panels were introduced and applied to a dataset from a sire breeding line in pigs. It was shown that imputation from low- to high-density marker panels is a promising strategy, even if the low-density panel contains less than 1000 markers. A number of 768 markers can be recommended, but 384 markers may be sufficient if at least one parent is genotyped at high-density. Careful selection of low-density markers is essential. The proposed regression method for obtaining unbiased estimates of the accuracy of genomic breeding values in a cross-validation setting showed promising results but has to be evaluated in more detail.

## Competing interests

The authors declare that they have no competing interests.

## Authors’ contributions

RW developed the methods and did the statistical analysis. RW and JB conceived and designed the experiment and wrote the paper. SP and KW generated the genotypes. JH and ET generated the conventional BLUP EBV. All authors read and approved the final manuscript.

## Supplementary Material

Additional file 1: Table S1 The table shows the genotype error rate and imputation accuracy per chromosome for the 384 marker panel. Click here for file

## References

[B1] Meuwissen THE Hayes BJ Goddard ME Prediction of total genetic value using genome-wide dense marker maps Genetics 2001 157 1819 1829 11290733 10.1093/genetics/157.4.1819 PMC1461589

[B2] Goddard ME Hayes BJ Mapping genes for complex traits in domestic animals and their use in breeding programmes Nat Rev Genet 2009 10 381 391 10.1038/nrg2575 19448663

[B3] Schaeffer LR Strategy for applying genome-wide selection in dairy cattle J Anim Breed Genet 2006 123 218 223 10.1111/j.1439-0388.2006.00595.x 16882088

[B4] Simianer H The potential of genomic selection to improve litter size in pig breeding programmes Proceedings of the 60th Annual Meeting of the European Association of Animal Production: 24–27 August 2009 2009 Barcelona: Wageningen Academic Publishers

[B5] Lillehammer M Meuwissen THE Sonesson AK Genomic selection for maternal traits in pigs J Anim Sci 2011 89 3908 3916 10.2527/jas.2011-4044 21841086

[B6] Ramos AM Crooijmans RP Affara NA Amaral AJ Archibald AL Beever JE Bendixen C Churcher C Clark R Dehais P Hansen MS Hedegaard J Hu ZL Kerstens HH Law AS Megens HJ Milan D Nonneman DJ Rohrer GA Rothschild MF Smith TP Schnabel RD Van Tassell CP Taylor JF Wiedmann RT Schook LB Groenen MA Design of a high density SNP genotyping assay in the pig using SNPs identified and characterized by next generation sequencing technology PLoS One 2009 4 e6524 10.1371/journal.pone.0006524 19654876 PMC2716536

[B7] Bennewitz J Wellmann R Neugebauer N Tholen E Wimmers K Results from genomic selection in Pietrain pig breeding Proceedings of the 62nd Annual Meeting of the European Federation of Animal Science: 29 August – 2 September 2001 2011 Stavanger: Wageningen Academic Publishers

[B8] Habier D Fernando RL Dekkers JCM Genomic selection using low-density marker panels Genetics 2009 182 343 353 10.1534/genetics.108.100289 19299339 PMC2674831

[B9] Hayes BJ Bowman PJ Daetwyler HD Kijas JW van der Werf JHJ Accuracy of genotype imputation in sheep breeds Animal Genet 2011 43 72 80 22221027 10.1111/j.1365-2052.2011.02208.x

[B10] Weigel KA Van Tassell CP O’Connell JR VanRaden PM Wiggans GR Prediction of unobserved single nucleotide polymorphism genotypes of Jersey cattle using reference panels and population-based imputation algorithms J Dairy Sci 2010 93 2229 2238 10.3168/jds.2009-2849 20412938

[B11] Lacy RC Analysis of founder representation in pedigrees: founder equivalents and founder genome equivalents Zoo Biol 1989 8 111 123 10.1002/zoo.1430080203

[B12] Marchini J Howie B Genotype imputation for genome-wide association studies Nat Rev Genet 2010 11 499 511 10.1038/nrg2796 20517342

[B13] Scheet P Stephens M A fast and flexible statistical model for large-scale population genotype data: applications to inferring missing genotypes and haplotypic phase Am J Hum Genet 2006 78 629 644 10.1086/502802 16532393 PMC1424677

[B14] Browning BL Browning SR A unified approach to genotype imputation and haplotype-phase inference for large data sets of trios and unrelated individuals Am J Hum Genet 2009 84 210 223 10.1016/j.ajhg.2009.01.005 19200528 PMC2668004

[B15] Daetwyler HD Wiggans GR Hayes BJ Woolliams JA Goddard ME Imputation of missing genotypes from sparse to high density using long-range phasing Genetics 2011 189 317 327 10.1534/genetics.111.128082 21705746 PMC3176129

[B16] Meuwissen T Goddard M The use of family relationships and linkage disequilibrium to impute phase and missing genotypes in up to whole-genome sequence density genotypic data Genetics 2010 185 1441 1449 10.1534/genetics.110.113936 20479147 PMC2927768

[B17] Hickey JM Kinghorn BP Tier B Wilson JF Dunstan N van der Werf JHJ A combined long-range phasing and long haplotype imputation method to impute phase for SNP genotypes Genet Sel Evol 2011 43 12 10.1186/1297-9686-43-12 21388557 PMC3068938

[B18] Garrick DJ Taylor JF Fernando RL Deregressing estimated breeding values and weighting information for genomic regression analyses Genet Sel Evol 2009 41 55 10.1186/1297-9686-41-55 20043827 PMC2817680

[B19] Amer PR Banos G Implications of avoiding overlap between training and testing data sets when evaluating genomic predictions of genetic merit J Dairy Sci 2010 93 3320 3330 10.3168/jds.2009-2845 20630248

[B20] Hill WG Robertson A Linkage disequilibrium in finite populations Theor Appl Genet 1968 38 226 231 10.1007/BF01245622 24442307

[B21] Browning SR Multilocus association mapping using variable-length Markov chains Am J Hum Genet 2006 78 903 913 10.1086/503876 16685642 PMC1474089

[B22] Browning SR Browning BL Rapid and accurate haplotype phasing and missing-data inference for whole-genome association studies by use of localized haplotype clustering Am J Hum Genet 2007 81 1084 1097 10.1086/521987 17924348 PMC2265661

[B23] Tortereau F Servin B Frantz L Megens HJ Milan D Rohrer G Wiedmann R Beever J Archibald AL Schook LB Groenen MAM A high density recombination map of the pig reveals a correlation between sex-specific recombination and GC content BMC Genomics 2012 13 586 10.1186/1471-2164-13-586 23152986 PMC3499283

[B24] Howie BN Donnelly P Marchini J A flexible and accurate genotype imputation method for the next generation of genome-wide association studies PLoS Genetics 2009 5 e1000529 10.1371/journal.pgen.1000529 19543373 PMC2689936

[B25] Vereijken ALJ Albers GAA Visscher J Imputation of SNP genotypes in chicken using a reference panel with phased haplotypes Proceedings of the 9th World Congress on Genetics Applied to Livestock Production: 1–6 August 2010; Leipzig 2010

[B26] Huang Y Hickey JM Cleveland MA Maltecca C Assessment of alternative genotyping strategies to maximize imputation accuracy at minimal cost Genet Sel Evol 2012 44 25 10.1186/1297-9686-44-25 22849718 PMC3436735

[B27] Wang C Habier D Peiris BL Wolc A Kranis A Watson KA Avendano S Garrick DJ Fernando RL Lamont SJ Dekkers JCM Accuracy of genomic prediction using an evenly spaced, low-density single nucleotide polymorphism panel in broiler chickens Poult Sci 2013 92 1712 1723 10.3382/ps.2012-02941 23776257

[B28] Goddard ME Genomic selection: prediction of accuracy and maximisation of long term response Genetica 2009 136 245 257 10.1007/s10709-008-9308-0 18704696

[B29] Daetwyler HD Villanueva B Woolliams JA Accuracy of predicting the genetic risk of disease using a genome-wide approach PLoS One 2008 3 e3395 10.1371/journal.pone.0003395 18852893 PMC2561058

[B30] Hayes BJ Daetwyler HD Bowman PJ Moser G Tier B Crump R Khatkar M Raadsma HW Goddard ME Accuracy of genomic selection: comparing theory and results Proc Assoc Advmt Anim Breed Genet 2009 18 34 37

[B31] Hayes BJ Visscher PM Goddard ME Increased accuracy of artificial selection by using realized relationship matrix Genet Res 2009 91 47 60 10.1017/S0016672308009981 19220931

