# Supplementary material for: Genomic selection using low density marker panels with application to a sire line in pigs
Source: Genet Sel Evol. 2013 Jul 29;45(1):28. doi: 10.1186/1297-9686-45-28 (PMC3750593; doi:10.1186/1297-9686-45-28)
Supplement: Additional file 1: Table S1 — The table shows the genotype error rate and imputation accuracy per chromosome for the 384 marker panel. [file 1297-9686-45-28-S1.doc]

Table S1: Genotype error rate and imputation accuracy per chromosome for the 384 marker panel.

| Chromosome | Error rate | Imputation accuracy | nSNP |
| --- | --- | --- | --- |
| 1 | 0.076 | 0.874 | 51 |
| 2 | 0.134 | 0.791 | 24 |
| 3 | 0.129 | 0.804 | 21 |
| 4 | 0.129 | 0.804 | 23 |
| 5 | 0.155 | 0.759 | 19 |
| 6 | 0.128 | 0.786 | 23 |
| 7 | 0.147 | 0.770 | 21 |
| 8 | 0.147 | 0.784 | 14 |
| 9 | 0.168 | 0.740 | 21 |
| 10 | 0.197 | 0.704 | 14 |
| 11 | 0.158 | 0.764 | 16 |
| 12 | 0.173 | 0.738 | 13 |
| 13 | 0.091 | 0.850 | 35 |
| 14 | 0.113 | 0.812 | 25 |
| 15 | 0.157 | 0.753 | 21 |
| 16 | 0.128 | 0.802 | 18 |
| 17 | 0.178 | 0.739 | 11 |
| 18 | 0.174 | 0.755 | 9 |
